# Maximizing Potential Applications of MAX Phases: Sustainable Synthesis of Multielement Ti_3_AlC_2_

**DOI:** 10.1021/acs.inorgchem.4c00648

**Published:** 2024-07-30

**Authors:** Filipa M. Oliveira, Nima Amousa, Amutha Subramani, Jan Luxa, Chenrayan Senthil, Zdeněk Sofer, Jesus Gonzalez-Julian

**Affiliations:** † Department of Inorganic Chemistry, Faculty of Chemical Technology, Prague University of Chemistry and Technology, Technická 5, Prague 6 166 28, Czech Republic; ‡ Chair of Ceramics, Institute of Mineral Engineering (GHI) RWTH Aachen University, Forckenbeckstrasse 33, Aachen 52074, Germany; § Department of Energy Engineering, Gyeonsang National University, Jinju-si 52725, Gyeongnam, South Korea

## Abstract

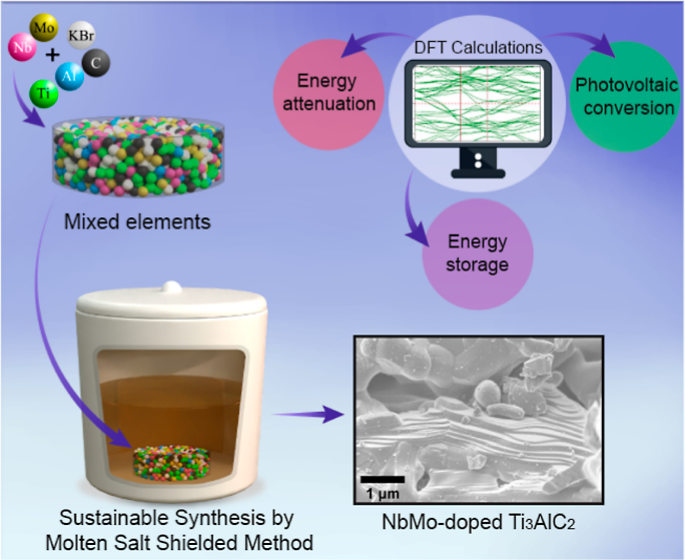

This study employs the molten-salt-shielded method to dope the Ti_3_AlC_2_ MAX phase with Nb and Mo, aiming to expand the intrinsic potential of the material. X-ray diffraction confirms the preservation of the hexagonal lattice structure of Ti_3_AlC_2_, while Raman and X-ray photoelectron spectroscopic analyses reveal the successful incorporation of dopants with subtle yet significant alterations in the vibrational modes and chemical environment. Scanning electron microscopy with energy-dispersive X-ray spectroscopy characterizations illustrate the characteristic layered morphology and uniform dopant distribution. Density functional theory simulations provide insights into the modified electronic structure, displaying changes in carrier transport mechanisms and potential increases in metallic conductivity, particularly when doping occurs at both the M and A sites. The computational findings are corroborated by the experimental results, suggesting that the enhanced material may possess improved properties for electronic applications. This comprehensive approach not only expands the MAX phase family but also tailors its functionality, which could allow for the production of hybrid materials with novel functionalities not present in the pristine form.

## Abstract 2

This study presents a novel Nb–Mo-doped Ti_3_AlC_2_ MAX phase synthesized via a sustainable molten-salt-shielded method. Characterization by inductively coupled plasma (ICP), scanning electron microscopy–energy-dispersive X-ray spectroscopy (SEM-EDS), Raman, and X-ray photoelectron spectroscopy (XPS) confirms successful metal doping of Ti_3_AlC_2_. Density functional theory (DFT) analysis elucidates the influence of Nb–Mo doping on electronic structure and transport properties, enhancing functionality. This study expands the MAX phase family, highlighting the potential for tailored material properties in different applications.

## 1 Introduction

In the world of cutting-edge materials, a remarkable fusion of metal and ceramic properties gives rise to a group of compounds called MAX phases. MAX phases, represented by the general formula M_*n+1*_AX_*n*_, where M represents an early transition metal, A is a 13 or 14-group element, X stands for carbon and/or nitrogen, and *n* ranges from 1 to 4, constitute a versatile and well-established family of layered ternary carbides and nitrides.^1,2^ The key advantages of these materials lie in their combination of metallic and ceramic properties, including good thermal and electrical conductivity, ease of machining, resistance to thermal shock, and remarkable damage tolerance.^3,4^ These exceptional attributes make them ideal candidates for a diverse range of applications such as spanning coatings,^5,6^ electrical contacts,^7^ catalysts for hydrogen storage,^8−11^ and concentrated solar power receivers^12^ even in harsh environments.^13^ Moreover, the role of MAX phases as precursors to their two-dimensional (2D) counterparts known as MXenes has sparked significant interest in more recent times.^14,15^

Among the diverse compositions of MAX phases, Ti_3_AlC_2_ stands out as one of the most extensively studied aluminum-containing carbides, attributed to its low-density, electric conductive properties,^16,17^ corrosion resistance,^17^ high-temperature oxidation resistance,^18,19^ and damage tolerance.^17^ The layered structure of Ti_3_AlC_2_ allows for customization and tailoring of its properties by introducing specific metal elements to suit various applications. For example, by doping Ti_3_AlC_2_ with selected transition metals, electromagnetic^20^ properties can be introduced and tailored, expanding its potential utility.

In the advancement of Ti_3_AlC_2_ MAX phase properties, our study focuses on niobium (Nb) and molybdenum (Mo) doping, driven by the unique characteristics of these transition metals. Niobium is selected for its excellent electrical conductivity, which is crucial for applications requiring efficient electron transport, for instance, in boosting the electrochemical performance of materials.^21,22^ Molybdenum, on the other hand, is known for its outstanding thermal stability and mechanical strength,^23,24^ characteristics that are beneficial in improving the high-temperature performance and durability of Ti_3_AlC_2_. The synergy of Nb and Mo in doping is hypothesized to not only retain but also augment the inherent properties of Ti_3_AlC_2_, such as its conductivity, corrosion resistance, and damage tolerance, being thus expected to broaden the scope of Ti_3_AlC_2_ applications.

Despite the potential of metal doping during the synthesis of Ti_3_AlC_2_, a gap persists between the promising aspects of this technique and the reported outcomes. Jun et al. reported on Fe-doped Ti_3_AlC_2_, showing the tunability of microwave absorption ability by varying the amount of Fe,^20^ while another report showcased electromagnetic interference shielding properties following Fe-doping and subsequent conversion to MXene.^25^ Mo doping was found to improve the lubricating properties and wear resistance of Ti_3_AlC_2_.^26^ Additionally, high-entropy structures based on Ti_3_AlC_2_ such as (TiVCrMo)_3_AlC_2_^27^ and (Mo_0.25_Cr_0.25_Ti_0.25_V_0.25_)_3_AlC_2_^28^ have also been prepared. Other investigations into metal doping of MAX phases, such as those focusing on bimetallic MAX phases like (Ti_1–*y*_Nb_*y*_)_2_AlC^29^ and (Ti_1–*x*_V_*x*_)_2_AlC,^30^ have further elucidated the structural and compositional effects of metal doping. Additionally, the incorporation of Fe, Co, Ni, and Mn in Vanadium MAX phases has also been reported.^31,32^ These studies emphasize the broader applicability and potential of metal doping in MAX phase compositions. Despite these advancements, considerable room exists for the exploration of metal doping and its potential applications.

Another important consideration involves the synthesis methods employed, often limited to the solid–solution reaction method, yielding only a few grams of the final product and requiring inert environments. However, an innovative and scalable alternative, the molten-salt-shielded synthesis (MS^3^) method^33^ has emerged. This approach recently enabled the synthesis of approximately 1 kg of a Ti_3_AlC_2_ MAX phase under sustainable conditions, in particular, in the air instead of using protective atmospheres such as vacuum and/or argon.^34^ Moreover, operating at lower temperatures and shorter processing times, the MS^3^ method reduces carbon footprint and greenhouse gas emissions, making it a promising advancement in the synthesis of ceramic materials that align with metal doping concepts and potential applications.

Herein, we present, for the first time, the synthesis of Ti_2.7_Nb_0.2_Mo_0.1_AlC_2_ MAX phase utilizing the MS^3^ technique. Structural analysis confirms the retention of the original hexagonal structure of Ti_3_AlC_2_, with ICP spectroscopy validating the elemental ratios. The characterization of Ti_2.7_Nb_0.2_Mo_0.1_AlC_2_ includes SEM–EDS, XPS, and Raman spectroscopy, all of which confirm the successful synthesis of this new MAX phase. Further, DFT analysis provides valuable insights into the modifications introduced by Nb–Mo metal doping, revealing the enhanced versatility of the Ti_3_AlC_2_ MAX phase properties.

## 2 Experimental Section

### 2.1 Synthesis of MAX Phases

Elemental powders of Ti (ThermoScientific, Germany, >99.5% pure, −325 mesh), Al (ThermoScientific, Germany, >99.5% pure, −325 mesh), C (Sigma-Aldrich, Germany, 99.5%, particle size <50 μm), Mo (Beijing Metallurgy and Materials Technology Co., Ltd., China, 99.9% pure, −300 mesh), and Nb (Beijing Metallurgy and Materials Technology Co., Ltd., China, 99.9% pure, −300 mesh) were weighed according to the ratios specified in Table S1 and mixed in a 500 mL high-density polyethylene bottle. Potassium bromide (KBr) from Lach-Ner, Czech Republic, was added in a 1:1 wt % ratio to the mixture. The constituents were ball-milled for 24 h using yttria-stabilized zirconia milling balls (⌀ 4.8–5.2 mm) in ethanol. Postmilling, the mixture underwent an hour of additional mixing using a multidirectional mixer (Turbula WAB, Switzerland). The resultant slurry was dried at 60 °C overnight and then pelletized under a 70 kN pressure using a steel mold (⌀ 30 mm) in a hydraulic press (Paul-Otto Weber, Germany). The pellet, placed in an alumina crucible (350 mL) and shielded with 300 g of KBr, was introduced in a furnace with the temperature ramped up at 2 °C/min to 1250 °C, maintained for 7 h, and cooled to room temperature at the same rate. The entire process was conducted in ambient air. Postsynthesis, the crucible was washed with hot deionized water (DIW) to dissolve the KBr, and the resultant MAX phase was recovered. This was followed by a 2 h boil in DIW and subsequent grinding in a mortar to achieve a fine-grained slurry. The slurry was then washed with hot DIW and vacuum-filtered. The final MAX powder was dried at 60 °C overnight and sieved through a 106 μm mesh by using a Vibratory Sieve Shaker AS 200 Basic (Retsch Technology, Germany). The same synthesis procedure was employed for Ti_3_AlC_2_, serving as a reference for characterization.

### 2.2 Materials Characterization

X-ray diffraction (XRD) patterns were acquired by using a Bruker D8 Advance instrument (Bruker AXS GmbH). The instrument was configured in Bragg–Brentano parafocusing geometry and employed a CuKα radiation source with λ = 0.15418 nm, operating at *U* = 40 kV and *I* = 40 mA. The XRD patterns were collected at room temperature over a 2θ range with a step size of 0.02°. Data analysis was conducted using the HighScore Plus 4.9 software package.

ICP optical emission spectroscopy (ICP-OES) was employed to determine the concentrations of Ti, Al, Nb, and Mo in the as-prepared metal-doped MAX phase using an ICP-715 spectrometer (Agilent Technologies, California, USA). The sample preparation for ICP-OES involves two steps: *first*, 50–60 mg of the MAX phase sample was mixed with a lithium borate melting agent in a platinum crucible. This mixture was then heated at 1050 °C for 5–10 min, forming a homogeneous molten glass. Lithium borate facilitates the transition of the MAX phase material from a solid to a glass-like state while also helping to eliminate carbon content. In the *second* step, the molten glass was cast into a polytetrafluoroethylene vessel containing about 200 mL of a 5 wt % HNO_3_ solution to digest the sample. The vessel was sealed and subjected to a two-stage heating process: first, they were heated for 5 min until reaching 180 °C, followed by 10 min of maintaining at 180 °C. Once cooled, the digested sample solution was transferred to a 25 mL volumetric flask, diluted to volume with ultrapure deionized water, and subsequently analyzed. This two-step process guarantees complete dissolution of the MAX phase material, allowing the quantitative determination of its elemental composition.

The morphology of the MAX phases was observed by SEM with a GeminiSEM 500 microscope from Zeiss (Germany) at a 2 kV acceleration voltage. An EDS detector from Oxford Instruments was used for element analysis and operated at a 15 kV acceleration voltage.

Raman spectroscopy was conducted utilizing an inVia Raman microscope (Renishaw, England) coupled with a Nd: YAG laser (532 nm, 50 mW) and a 20× objective. The data were acquired directly from the powder samples within the Raman range of 100–800 cm^–1^ at room temperature. The measurements involved an exposure time of 10 s, 10 accumulations, and an applied power of 1.25%.

HR-XPS was performed using a Phoibos 100 (Specs, Germany) instrument with a monochromatic Al X-ray radiation source (1486.7 eV). The samples were placed on a conductive carbon tape and compensated with a flood gun to yield a C 1s peak at 285 eV. Wide-scan surveys were collected, with subsequent HR scans for Ti 2p, Al 2p, Mo 3d, and Nb 3d core levels. The Casa XPS software package was employed to perform quantification and curve fitting on the core-level spectra using Shirley-type backgrounds.

### 2.3 Computer Modeling

Structural and electronic calculations were performed using DFT as fulfilled in the Cambridge serial total energy package. For the structural optimization, the ultrasoft pseudopotential was used to simulate the interaction between electron and ion cores in geometric structure optimization and single point energy calculation, and the Pardew Burke Ernzerhof (PBE) with a generalized gradient approximation (GGA) was used to describe the exchange–correlation function. The scissors value is used to correct for the calculated band gap value. The cutoff energy was set as 360 eV, and the *K* point was set to 1 × 1 × 1. The convergence threshold for SCF tolerance is 2 × 10^–6^ eV/atom between two electronic steps, and the maximum force upon each atom is less than 0.01 eV/Å.

No uncommon hazards are noted. During the high-temperature synthesis, safety procedures must be followed as gases might be released and furnace components become extremely hot as the temperature reaches 1250 °C.

## 3 Results and Discussion

### 3.1 Synthesis and Characterization of MAX Phases

A metal-doped Ti_3_AlC_2_ MAX phase was successfully synthesized using the MS^3^ method under reaction conditions detailed in Table S1. KBr was used as a molten salt, enabling synthesis in air. Figure S1 illustrates the visual progression of the materials through various stages of the process, from the initial pellet (Figure S1a) to after synthesizing (Figure S1b) and washing (Figure S1c), resulting in the metal-doped Ti_3_AlC_2_ MAX phase as a fine powder (Figure S1d).

Following synthesis, ICP spectroscopy was conducted to determine the molar ratios of elements within the material, yielding Ti/Al/Nb: Mo at 2.7:1:0.2:0.1. These results are consistent with the reaction conditions specified in Table S1, leading to the chemical designation of the doped phase as Ti_2.7_Nb_0.2_Mo_0.1_AlC_2_.

**Figure 1:**
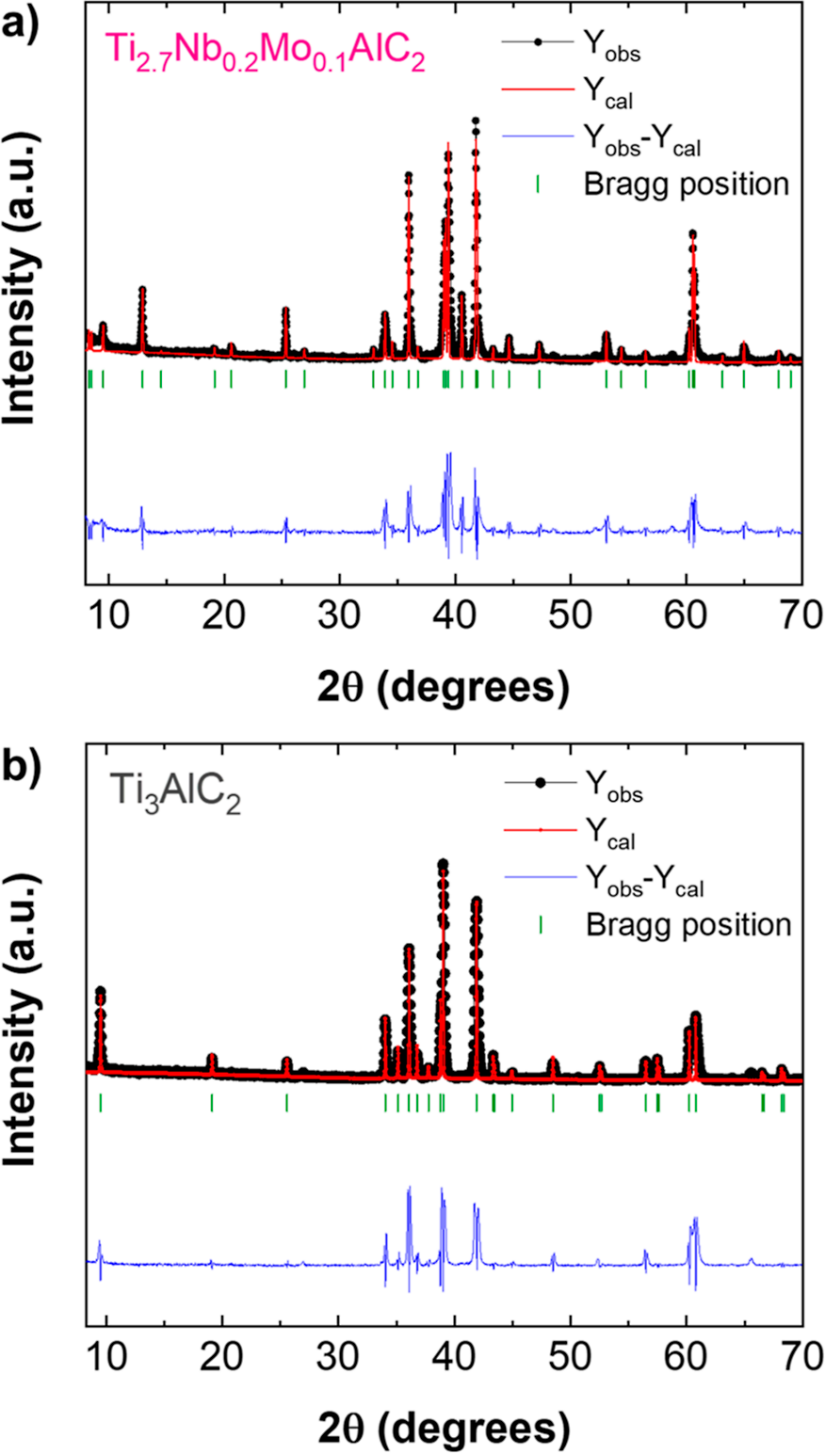
XRD characterization of (a) Ti_2.7_Nb_0.2_Mo_0.1_AlC_2_ and (b) Ti_3_AlC_2_ MAX phases with respective Rietveld refinement.

The XRD pattern of Ti_2.7_Nb_0.2_Mo_0.1_AlC_2_ is presented in Figure 1a. Characteristic diffraction peaks at 9.496, 33.900, 41.766, and 60.571° correlate with the (002), (101), (105), and (110) planes of Ti_3_AlC_2_ (Figure 1b), respectively, confirming the hexagonal *P* 6_3_/*mmc* space group (no. 194) typical of the 312 MAX phase.^35^ Additional peaks indicate the presence of impurities (Figure S2a): TiNbAlC at 12.896° (PDF 04-005-0366), Al_2_O_3_ at 25.304° (PDF-00-046-1212)—likely from Al powder oxidation—TiC at 33.900° (PDF-03-065-8805), and metallic Mo at 40.525° (PDF-04-005-7149). Rietveld refinement, based on space group *P 6*_3_*/mmc* (no. 194), was performed for both Ti_2.7_Nb_0.2_Mo_0.1_AlC_2_ (Figure 1a) and pristine Ti_3_AlC_2_ (Figure 1b). The lattice parameters of the Ti_2.7_Nb_0.2_Mo_0.1_AlC_2_, *a* = 3.0716 Å and *c* = 18.624 Å, closely align with the hexagonal structure of the parent Ti_3_AlC_2_, consistent with reports on the 312 MAX phase.^35^ Furthermore, a rational comparison of the experimental XRD patterns of Ti_3_AlC_2_ and Ti_2.7_Nb_0.2_Mo_0.1_AlC_2_ shown in Figure S2b reveals no significant peak broadening or shifts upon doping with Nb and Mo atoms, indicating that the lattice parameters remain similar.

**Figure 2:**
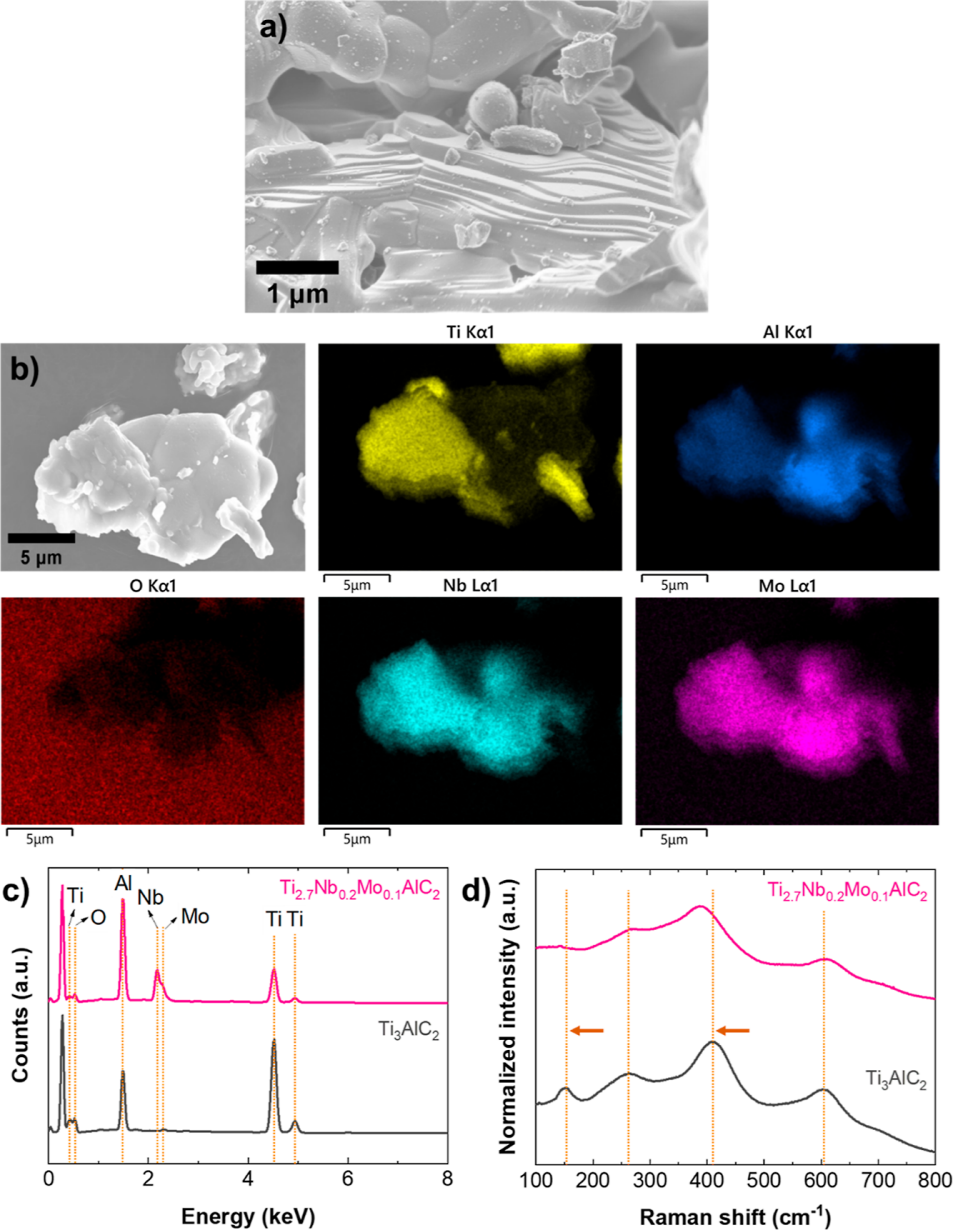
Characterization of Ti_2.7_Nb_0.2_Mo_0.1_AlC_2_: (a) SEM micrographs and (b) elemental mapping of elements. Comparative (c) EDS and (d) Raman spectra of doped and pristine Ti_3_AlC_2_.

The SEM micrograph in Figure 2a shows the characteristic layered morphology of the MAX phases. The elemental composition and a relatively homogeneous distribution confirmed by EDS analysis in Figure 2b validate the incorporation of Nb and Mo elements into Ti_3_AlC_2_. The presence of Nb and Mo peaks in the EDS spectrum (Figure 2c) further confirms the inclusion of these dopants in the Ti_3_AlC_2_ structure. For comparative purposes, Figure S3 shows SEM and EDS elemental mapping of the pristine Ti_3_AlC_2_ MAX phase.

**Figure 3:**
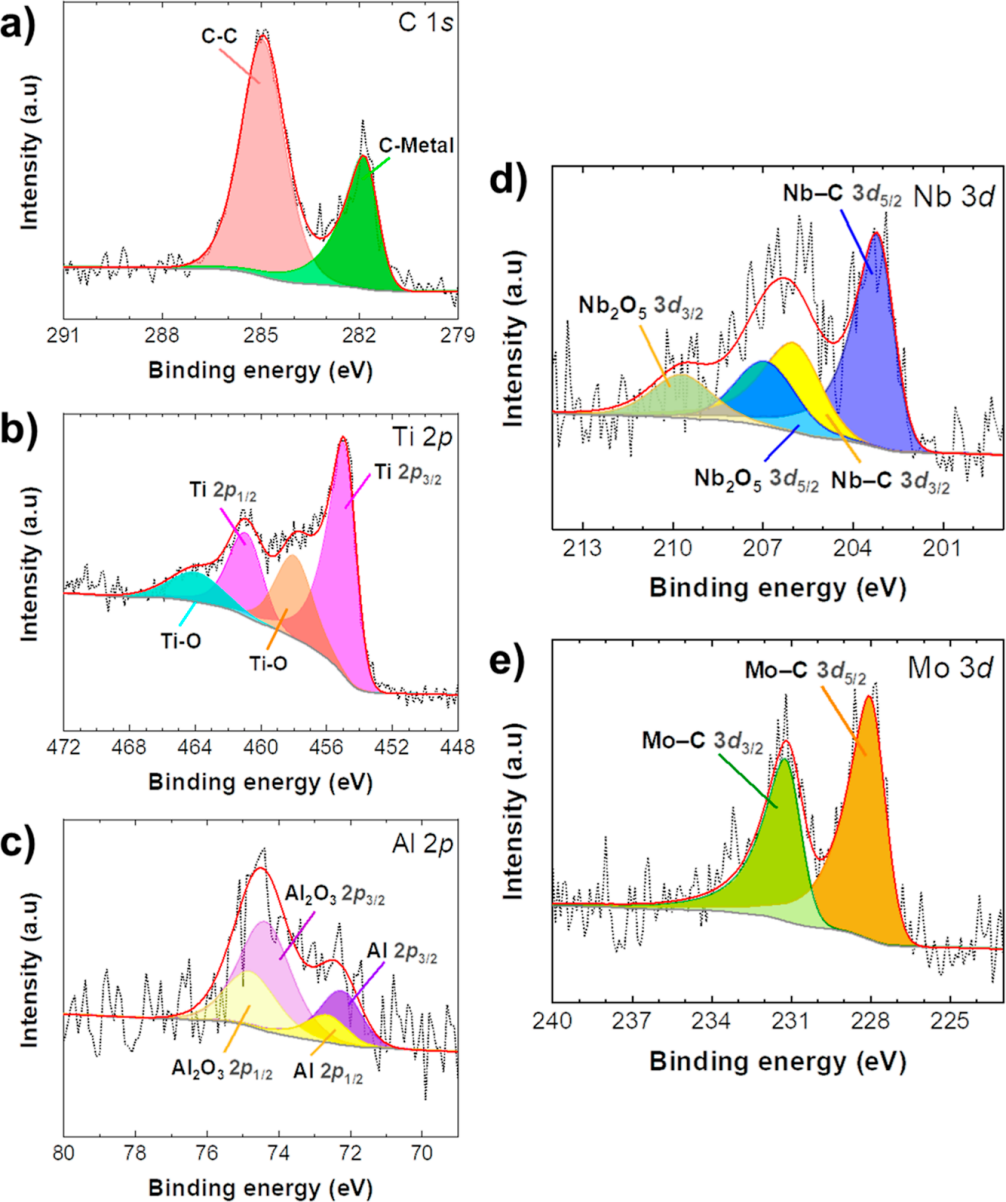
HR-XPS characterization of Ti_2.7_Nb_0.2_Mo_0.1_AlC_2_: (a) C 1s, (b) Ti 2p, (c) Al 2p, (d) Nb 3d, and (e) Mo 3d core levels. The red straight line represents the fitting, the dark short dotted line represents the experimental result, and the gray straight line represents the background.

The surface elemental composition and chemical states of the synthesized MAX phases were investigated by XPS analysis. The XPS-wide survey spectra for both Ti_3_AlC_2_ and Ti_2.7_Nb_0.2_Mo_0.1_AlC_2_, shown in Figure S4a,b, respectively, confirm the presence of the constituent elements. Detailed peak fitting results from these analyses are compiled in Table S2. High-resolution (HR) XPS spectra for core levels of Ti_2.7_Nb_0.2_Mo_0.1_AlC_2_ are exhibited in Figure 3, while the pristine Ti_3_AlC_2_ HR-XPS spectra are available for comparison in Figure S5. Within the C 1s HR spectrum (Figure 3a), the identification of C–C bonds from adventitious carbon and *C*-metal bonds is evident. The peaks corresponding to C–Metal bonds were deconvoluted with an asymmetric peak shape, as previously reported,^36^ indicative of the metallic conductive nature prevalent in materials like MXenes.^37^ Both Ti 2p (Figure 3b) and Al 2p (Figure 3c) HR spectra reveal dual components: one concerning the MAX phase and another to a surface oxide layer, with precise peak positions listed in Table S2. Such surface oxidation is an anticipated phenomenon due to the strong affinity toward oxygen by these metals.^36^ For the Ti_2.7_Nb_0.2_Mo_0.1_AlC_2_ sample, HR core-level spectra exhibit asymmetric peaks that can be ascribed to Nb–C (Figure 3d) and Mo–C bonds (Figure 3e), aligning with binding energies reported in the literature.^38^ Noteworthy is that an additional peak pair is detected in the Nb 3d region (Figure 3d), at higher binding energies, which is attributed to the surface oxidation products of Nb, specifically Nb_2_O_5_, indicating its higher susceptibility to oxidation. Overall, the XPS results validate the effective incorporation of both Mo and Nb into the structure of the Ti_3_AlC_2_ MAX phase, as made evident by the distinct presence of their respective chemical states.

The Raman spectra in Figure 2d provide further insights into the structural modifications that Nb–Mo doping introduces to the Ti_3_AlC_2_ MAX phase. Ti_2.7_Nb_0.2_Mo_0.1_AlC_2_ exhibits peaks at 143.4 (ω_1_), 264.8 (ω_2_), 386.8 (ω_3_), and 604.8 (ω_4_) cm^–1^. These peaks can be assigned to in-plane vibrations of Al–Ti (*E*_*g*(1)_), carbon (*A*_1*g*_), and oxygen bonds (*A*_1*g*_), respectively, while ω_4_ is associated with Ti–C vibrations (*E*_*g*(2)_).^39^ Comparative analysis with pristine Ti_3_AlC_2_ shows that ω_2_ and ω_4_ remain relatively unchanged in terms of peak positions, indicating that some vibrational characteristics are preserved upon doping (Figure 2d–Table S3). However, the observed downshifts for ω_1_ (by 8.1 cm^–1^) and ω_3_ (by 22.1 cm^–1^) suggest significant modifications in the local bonding environment, likely a consequence of the integration of Nb and Mo. Furthermore, as noted in the XPS results, the additional peak pair in the Nb 3d region, which is attributed to the surface oxidation products of Nb (Figure 3d–Table S2), confirms the incorporation of Nb. In the Raman spectrum, this downshift of ω_3_ associated with the oxygen bonds aligns with this observation, contributing to the alteration of the local bonding environment. Despite these changes, the preservation of characteristic frequencies of the pristine Ti_3_AlC_2_ MAX phase, with no new peaks, supports the idea that the structural integrity of the MAX phase remains intact.

To assess the long-term stability and evaluate the oxidation effects on the Ti_2.7_Nb_0.2_Mo_0.1_AlC_2_, which was stored under ambient conditions, additional XPS measurements were conducted 10 months postsynthesis (Figure S6 and Table S2). These results show an increase in the surface oxidation: TiO_2_ concentration rose from 27.3 to 42.4 at. %, Nb oxide from 35.4 to 57.3 at. %, and Al_2_O_3_ from 74.6 to 84.5 at. %. Molybdenum now exhibits the presence of MoO_2_ at 49 at. % and MoO_3_ at 18.5 at. %. Despite these changes, the asymmetric peak shapes characteristic of the Ti_3_AlC_2_ MAX phase remain, suggesting that the doped material properties are largely preserved despite surface oxidation.

Additionally, EDS analysis was performed to further investigate the elemental distribution after the same period. The results confirm the distribution of elements within the sample, with the elemental spectrum nearly overlapping that of the as-synthesized sample (Figure S7). This almost identical elemental composition as seen in the EDS data confirms that the intrinsic properties of Ti_2.7_Nb_0.2_Mo_0.1_AlC_2_ are maintained even after extensive periods, highlighting its potential for long-term applications.

### 3.2 DFT Calculations

To investigate the potential effects of Nb–Mo doping on Ti_3_AlC_2_, we conducted DFT simulations. Using refined data for Ti_3_AlC_2_ (lattice constants *a* = *b* = 3.07159 Å and *c* = 18.62398 Å, space group of *P* 6_3_/*mmc*, no. 194), Figure 1b, we constructed a crystal structure model (Figure S8). In order to observe differences in the electronic structure and transport carriers in Nb–Mo-doped Ti_3_AlC_2_, computational modeling suggested a dopant percentage of 0.2 for both Nb and Mo. Hence, two site doping strategies were considered: (i) (Ti_0.6_M1_0.2_M2_0.2_)_3_AlC_2_ (M1=Nb, M2=Mo) by M site doping and (ii) (Ti_0.8_M_0.2_)_3_(A_0.2_Al_0.8_)C_2_ (M=Nb, A=Mo) by doping at both M and A sites. The possibility of metal doping in the M–A site has not been considered in the traditional definition of MAX phases, and our work aims to shed light on the possibilities of such doping configurations.

**Figure 4:**
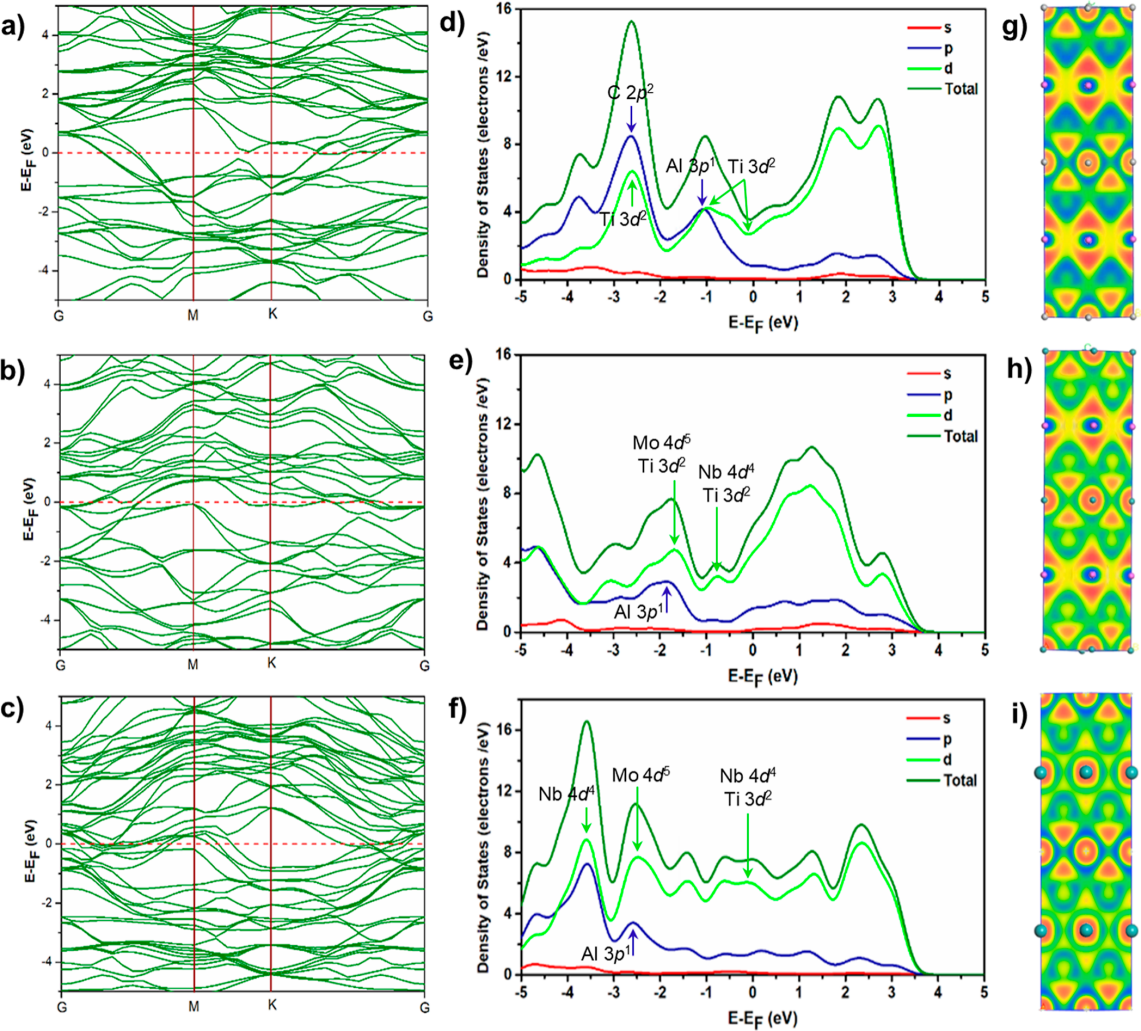
a–c) Electronic band structure, (d–f) partial and total density of states, and (g–i) electron localization function of the parent Ti_3_AlC_2_ (top), (Ti_0.6_M1_0.2_M2_0.2_)_3_AlC_2_ (M1=Nb, M2=Mo) by M-doped site (center), and (Ti_0.8_M_0.2_)_3_(A_0.2_Al_0.8_)C_2_ (M=Nb, A=Mo) by both M- and A-doped sites (bottom).

Given the significance of band structure in understanding the electronic properties of materials, Figure 4a–c depicts the electronic band structure of the parent Ti_3_AlC_2_ (Figure 4a), Ti_3_AlC_2_ via M doping site (Figure 4b), and Ti_3_AlC_2_ doped at M and A sites (Figure 4c) at high symmetry locations. The top of the valence band maximum and bottom of the conduction band minimum overlap and diverge at the Brillouin zone symmetry point responsible for zero band gap for the parent, validating metallic behavior consistent with previously reported theory results,^40,41^ and Nb–Mo doped Ti_3_AlC_2_. The normal metallic behavior with bands crossing the Fermi level along various directions results in a finite DOS −3.74 states/eV cell for parent Ti_3_AlC_2_,^41^ 6.61 states/eV for (Ti_0.6_M1_0.2_M2_0.2_)_3_AlC_2_, and 7.74 states/eV for (Ti_0.8_M_0.2_)_3_(A_0.2_Al_0.8_)C_2_ at the Fermi level. As a result, Ti_3_AlC_2_ and Nb–Mo doped Ti_3_AlC_2_ exhibit metallic properties such as metallic conductivity increases in the Nb–Mo doped Ti_3_AlC_2_. The band structures also reveal a strongly anisotropic feature with a smaller energy dispersion along the *c*-axis. The anisotropy of the band structure near and below *E*_F_ indicates that electrical conductivity is lower in the *c* direction compared to the ab plane for single-crystal Ti_3_AlC_2_ and M site-doped Ti_3_AlC_2_. However, in the case of both M and A site doping (Figure 4c), the bands become denser in the valence and conduction bands and appear to be isotropic near the Fermi level. As a result, the electrical conductivity will be maximum compared to that of the parent and M site-doped Ti_3_AlC_2_.

Then, density of states (DOS) calculations (Figure 4d–f), including both total density of states and partial density of states, were conducted to provide a comprehensive understanding of the electron contributions in both the valence and conduction bands for Ti_3_AlC_2_- and Nb–Mo-doped counterparts. Pseudoatomic calculations of valence electrons were performed. For Ti_3_AlC_2_, these calculations considered the valence electrons of C (2s^2^ 2p^2^), Al (3s^2^ 3p^1^), and Ti (3s^2^ 3p^6^ 3d^2^ 4s^2^). In the case of metal-doped Ti_3_AlC_2_, valence electron calculations were extended to include Nb (4s^2^ 4p^2^ 4d^4^ 5s^1^) and Mo (4s^2^ 4p^6^ 4d^5^ 5s^1^) in addition to C, Al, and Ti. Our findings indicate that the highest valence band (VB1, VB2, and VB3) states have Ti 3d^2^-state and Al 3p^1^-state electron contributions, with a maximum d electron contribution. In contrast, the lowest valence band (VB4) is mainly shaped by 2p^2^-state electrons of p, resulting in minimal C atom contributions near the Fermi level. However, in conduction bands, Ti-3d^2^ orbitals play a crucial role in determining the electronic properties.

A strong hybridization is observed between Ti 3d and Al 3p orbitals, particularly in the highest valence bands near the Fermi level, which plays a significant role in determining the metallic behavior of the material. When considering M site doping (Figure 4e) and both M and A site doping (Figure 4f) in Ti_3_AlC_2_, Al 3p^1^ state electron contributions decrease compared to the parent Ti_3_AlC_2_ (Figure 4d). In these doped cases, both Ti 3d and Nb–Mo 4d electron contributions are enhanced near the Fermi level and extended to the conduction band, which results in a significant increase in the magnetic behavior of the system, as depicted in Figure 4d–f.

Electron localization function (ELF) analysis offers a direct measure of electron localization, quantifying it within a range of 0 to 1. In Figure 4g–i, ELF domains are visually represented by isosurfaces with an isovalue of 0.5. These visualizations allow us to observe changes in electron localization within the context of parent Ti_3_AlC_2_ (Figure 4g), M site-doped Ti_3_AlC_2_ (Figure 4h), and the case of both M and A site-doped Ti_3_AlC_2_ (Figure 4i). For both M and A site-doped Ti_3_AlC_2_ (Figure 4i), these changes are notably pronounced. Doping at the M and A sites induces a substantial deformation of both the valence and core domains, bringing them closer and creating interconnected pathways that facilitate electron mobility. In contrast, in the absence of doping (Figure 4g), the domains remain distinct with no interconnected electron pathways. The blue regions, particularly the dark blue areas, indicate a decrease in electron localization, while the red half-moon regions signify localized electrons. When electrons are introduced at the A site, they exhibit a preference for localization at the outermost edges of the region. As depicted in Figure 4a–c, charge transfer occurs between Ti–Al bonds, yet the ELF values in Figure 4g–i unveil a localization region between the Ti–Al bonds. Furthermore, the bonding between Nb and Mo 4d orbitals strengthens when doped with Ti 3d orbitals at the A site.

**Figure 5:**
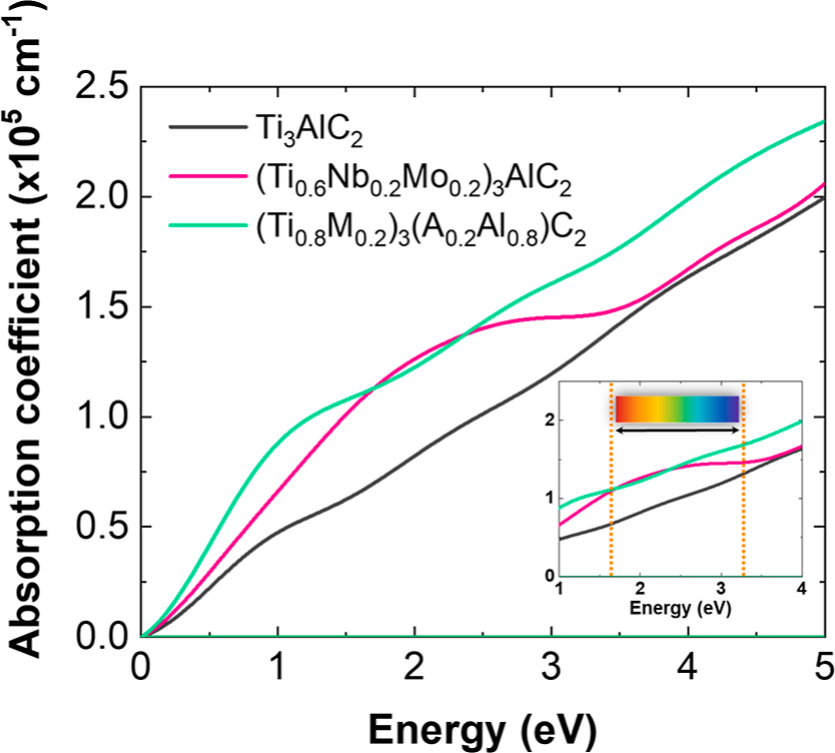
Absorption spectrum of Ti_3_AlC_2_ and Ti_3_AlC_2_ doped at M and M–A sites.

Lastly, to gain insights into the optical characteristics of Ti_3_AlC_2_ and Nb–Mo-doped Ti_3_AlC_2_, we employed the GGA with the PBE functional. As shown in Figure 5, the absorption coefficient as a function of the energy is presented. At first glance, it is observed that the materials effectively absorb light within the measured energy range, as the absorption coefficient exceeds 10^4^ cm^–1^.^42^ NbMo-doped compositions exhibit higher absorption coefficients compared to Ti_3_AlC_2_, with values at 4.98 eV of 1.98 × 10^5^ cm^–1^ for Ti_3_AlC_2_, 2.0 × 10^5^ cm^–1^ for (Ti_0.6_M1_0.2_M2_0.2_)_3_AlC_2_, and 2.3 × 10^5^ cm^–1^ for (Ti_0.8_M_0.2_)_3_(A_0.2_Al_0.8_)C_2_. This observation further supports the idea that metal doping enhances the interaction of Ti_3_AlC_2_ with light. With a practical application perspective in mind, a detailed analysis within the energy range of 1.63 to 3.26 eV (inset of Figure 5) was conducted, corresponding to the visible light region of the electromagnetic spectrum.^43^ In this range, M-site-doped Ti_3_AlC_2_ exhibits an absorption coefficient ranging from 1.09 × 10^5^ cm^–1^ to 1.46 × 10^5^ cm^–1^, while both M and A-site-doped Ti_3_AlC_2_ range from 1.11 × 10^5^ cm^–1^ to 1.68 × 10^5^ cm^–1^.

The pronounced increase in absorption for (Ti_0.6_M1_0.2_M2_0.2_)_3_AlC_2_ is attributed to significant hybridization of Nb 4d^4^ with Ti 3d^2^, alongside Mo 4d^5^ and Ti 3d^2^ electron interaction in the energy range from 0 to −2 eV, which enhances optical properties notably around 2.2 eV as seen in the DOS plot (Figure 4e). On the other hand, the (Ti_0.8_M_0.2_)_3_(A_0.2_Al_0.8_)C_2_ composition demonstrates maximum d electron contribution across the lower energy region of the conduction band from 0 eV to −4.0 eV (Figure 4f), amplifying optical absorption, particularly at photon energies above approximately ≈4.0 eV (Figure 5). These findings suggest that both (Ti_0.6_M1_0.2_M2_0.2_)_3_AlC_2_ and (Ti_0.8_M_0.2_)_3_(A_0.1_Al_0.9_)C_2_ can be applied in the UV–vis range, while (Ti_0.8_M_0.2_)_3_(A_0.2_Al_0.8_)C_2_ extends its applicability toward the infrared and ultraviolet ranges. This analysis sheds light on the potential applications of these materials in the design of photoelectronic devices where efficient light absorption is a primary requirement, such as photovoltaic conversion.

The suggested possibilities for efficient charge transport in Nb–Mo-doped Ti_3_AlC_2_ can significantly impact applications where such transport is essential. This impact varies depending on whether the doping occurs on the M site—(Ti_0.6_M1_0.2_M2_0.2_)_3_AlC_2_—or involves both M and A site doping—(Ti_0.8_M_0.2_)_3_(A_0.2_Al_0.8_)C_2_. However, it is important to note that Ti_3_AlC_2_ serves as a precursor for the synthesis of one of the most extensively studied MXene, Ti_3_C_2_T_*x*_. In this context, metal doping at the A site may not be advantageous as it could be removed during the etching process of the MAX phase. Therefore, choosing the relevant site for doping is critical when designing the synthesis of the desired MAX phase. Nonetheless, it is worth noting that our findings underscore the tunability of Ti_3_AlC_2_ MAX phase properties via Nb and Mo doping at relevant sites, making them adaptable to specific requirements across a wide range of applications, including electronics, energy storage, and conversion, as well as in the domains of electromagnetic energy dissipation and absorption.

## 4 Conclusions

In this study, we have successfully synthesized a new metal-doped Ti_3_AlC_2_ MAX phase using the sustainable MS^3^ method. The new MAX phase, confirmed to crystallize with the space group *P* 6_3_/*mmc* (194), has been further characterized through ICP analysis to have the specific stoichiometry of Ti_2.7_Nb_0.2_Mo_0.1_AlC_2_, confirming the dopant composition of Nb = 0.2 and Mo = 0.1. Raman and XPS analyses confirmed the integration of the dopants and revealed changes in the local bonding environment within Ti_3_AlC_2_. DFT analysis has provided valuable insights into the influence of Nb–Mo doping on the electronic structure and transport properties of the Ti_3_AlC_2_ MAX phase. We explored two doping strategies: selective M site doping and doping at both the M and A sites. These approaches have unveiled the capacity to adjust the MAX phase properties, aligning them with the demands of various applications. Notably, the DFT calculations suggest that such doping can enhance metallic conductivity and potentially modify magnetic and optical responses, as evidenced by increased absorption coefficients, especially in the UV–vis range. Our work not only extends the MAX phase family but also offers knowledge into the means to fine-tune their properties, thereby expanding their potential for a wide array of technological applications.

## Supporting Information Available

Supporting digital photographs and comparison of experimental XRD pattern of produced MAX phases; SEM micrograph and elemental mapping of elements of Ti_3_AlC_2_ MAX phase; XPS wide survey of Ti_3_AlC_2_ and Ti_2.7_Nb_0.2_Mo_0.1_AlC_2_ MAX phases; HR-XPS spectra for different core levels of Ti_3_AlC_2_ MAX phase and Ti_2.7_Nb_0.2_Mo_0.1_AlC_2_, characterized 10 months postsynthesis; EDS analysis of Ti_2.7_Nb_0.2_Mo_0.1_AlC_2_, characterized 10 months postsynthesis; crystal model structure of Ti_3_AlC_2_ with M and A site substitutions by Nb and Mo transition metals; and reaction conditions, XPS, and Raman spectroscopy data (PDF)
