# Supplementary material for: Maximizing Potential Applications of MAX Phases: Sustainable Synthesis of Multielement Ti3AlC2
Source: Inorg Chem. 2024 Jul 30;63(32):14851–9. doi: 10.1021/acs.inorgchem.4c00648 (PMC11323249; doi:10.1021/acs.inorgchem.4c00648)
Supplement: Supplementary file 1 — ic4c00648_si_001.pdf [file ic4c00648_si_001.pdf]

Supporting Information

Maximizing Potential Applications of MAX phases: Sustainable Synthesis of Multielement Ti_3AlC_2

Filipa M. Oliveira, [ⱡ*] *Nima Amousa,* [‡] *Amutha Subramani,* [ⱡ] *Jan Luxa,* [ⱡ] *Chenrayan Senthil,* [§] *Zdeněk Sofer,* [ⱡ*] *Jesus Gonzalez-Julian* [‡*]

[ⱡ] Department of Inorganic Chemistry

Faculty of Chemical Technology, University of Chemistry and Technology Prague

Technická 5, 166 28 Prague 6, Czech Republic

[‡] Chair of Ceramics, Institute of Mineral Engineering (GHI)

RWTH Aachen University

Forckenbeckstrasse 33, 52074 Aachen, Germany

[§] Department of Energy Engineering

Gyeonsang National University

Jinju-si, Gyeongnam, 52725, South Korea

Corresponding authors:

* Filipa M. Oliveira: filipa.oliveira@vscht.cz

* Zdeněk Sofer: zdenek.sofer@vscht.cz

* Jesus Gonzalez-Julian: gonzalez@ghi.rwth-aachen.de

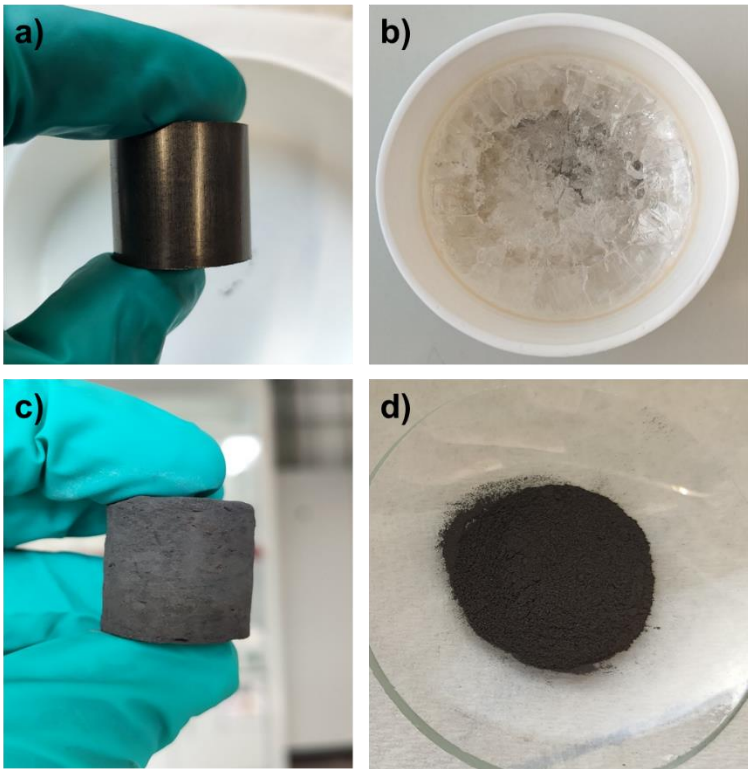

Figure S1. Digital photographs illustrating the visual characteristics of materials at different synthesis stages of $Ti_{2.7}Nb_{0.2}Mo_{0.1}AlC_2$ MAX phase: a) pellet before synthesis, b) pellet shielded by KBr after synthesis, c) MAX pellet after synthesis and washed with boiled water, d) synthesized material powder.

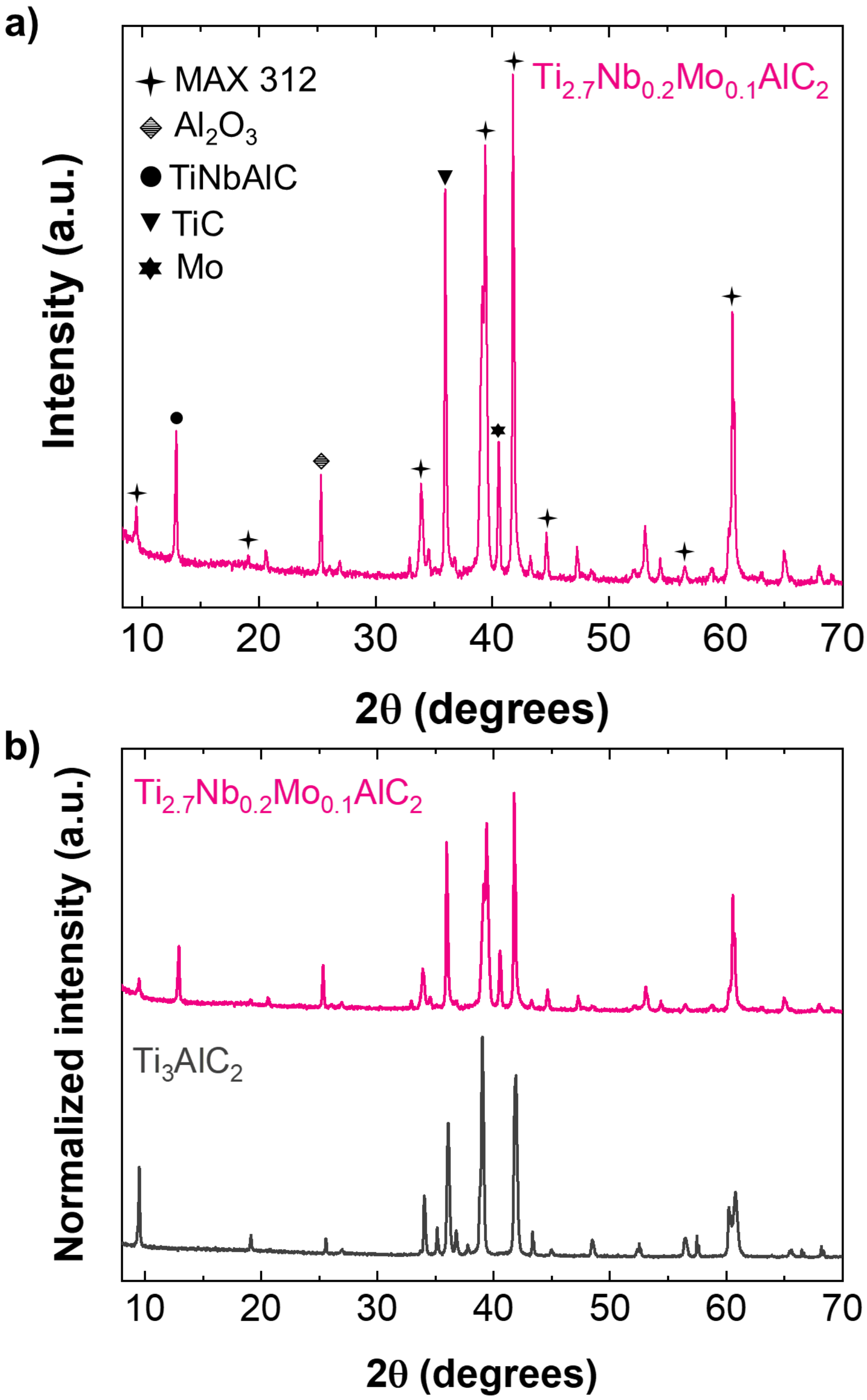

Figure S2. XRD characterization: a) experimental XRD pattern of $Ti_{2.7}Nb_{0.2}Mo_{0.1}AlC_2$ MAX phase. For incorporation of Nb and Mo, the XRD pattern shows that the main product is a 312 MAX phase with impurities of Al_2O_3, TiNbAlC, TiC and Mo. b) Comparison of experimental XRD patterns of of Ti_3AlC_2 and $Ti_{2.7}Nb_{0.2}Mo_{0.1}AlC_2$ MAX phase.

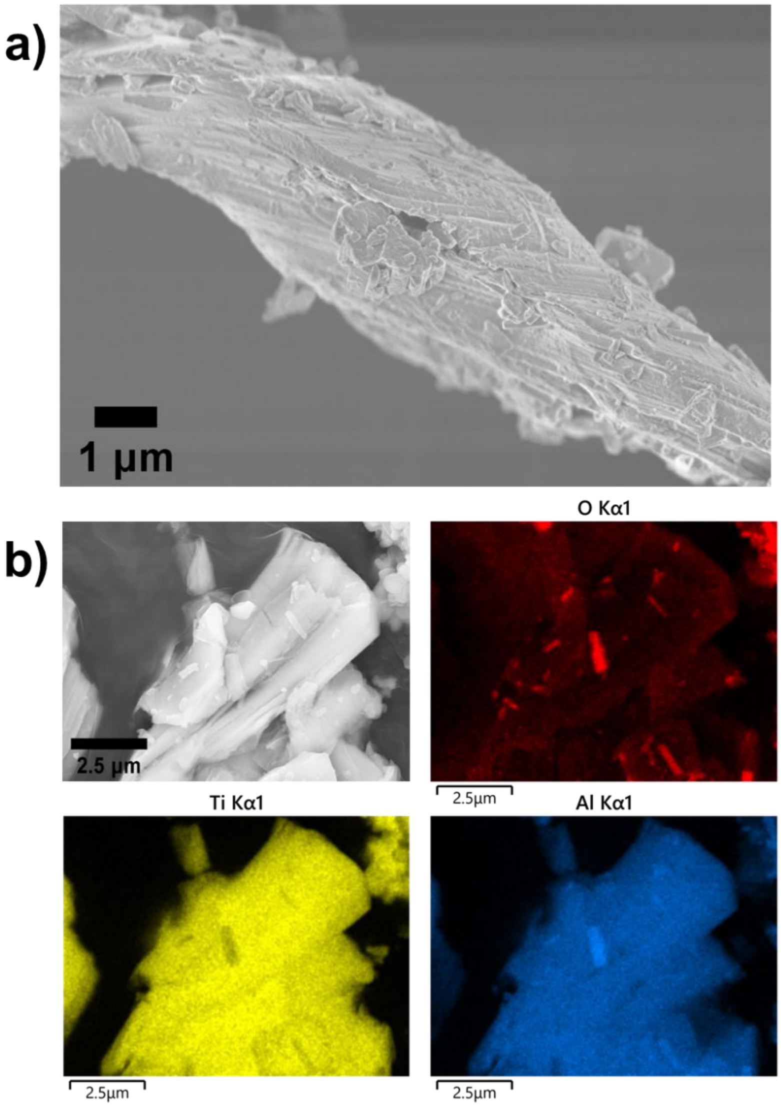

Figure S3. a) SEM micrograph and b) elemental mapping of elements for Ti_3AlC_2 MAX phase.

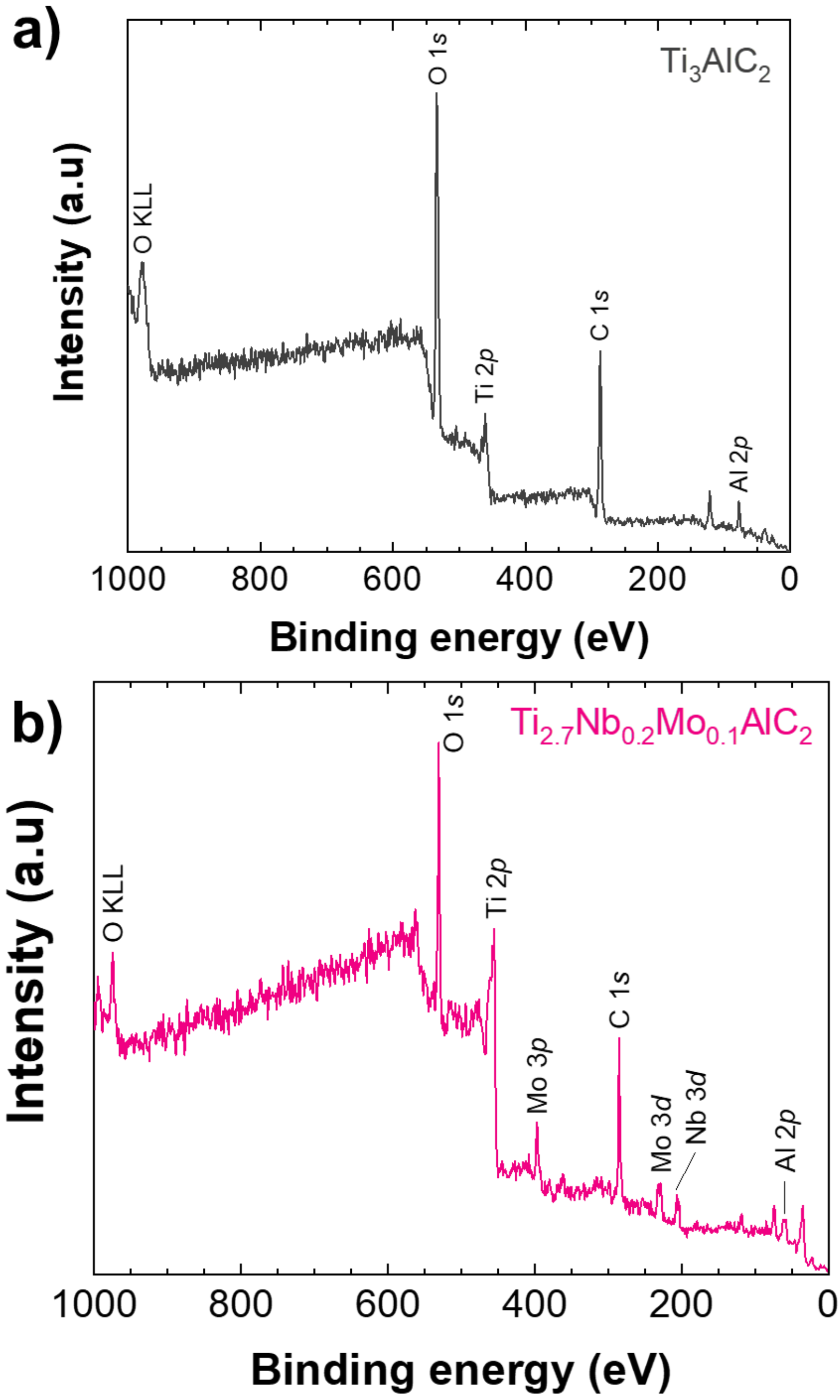

Figure S4. XPS wide survey spectra of a) Ti_3AlC_2 and b) $Ti_{2.7}Nb_{0.2}Mo_{0.1}AlC_2$ MAX phases.

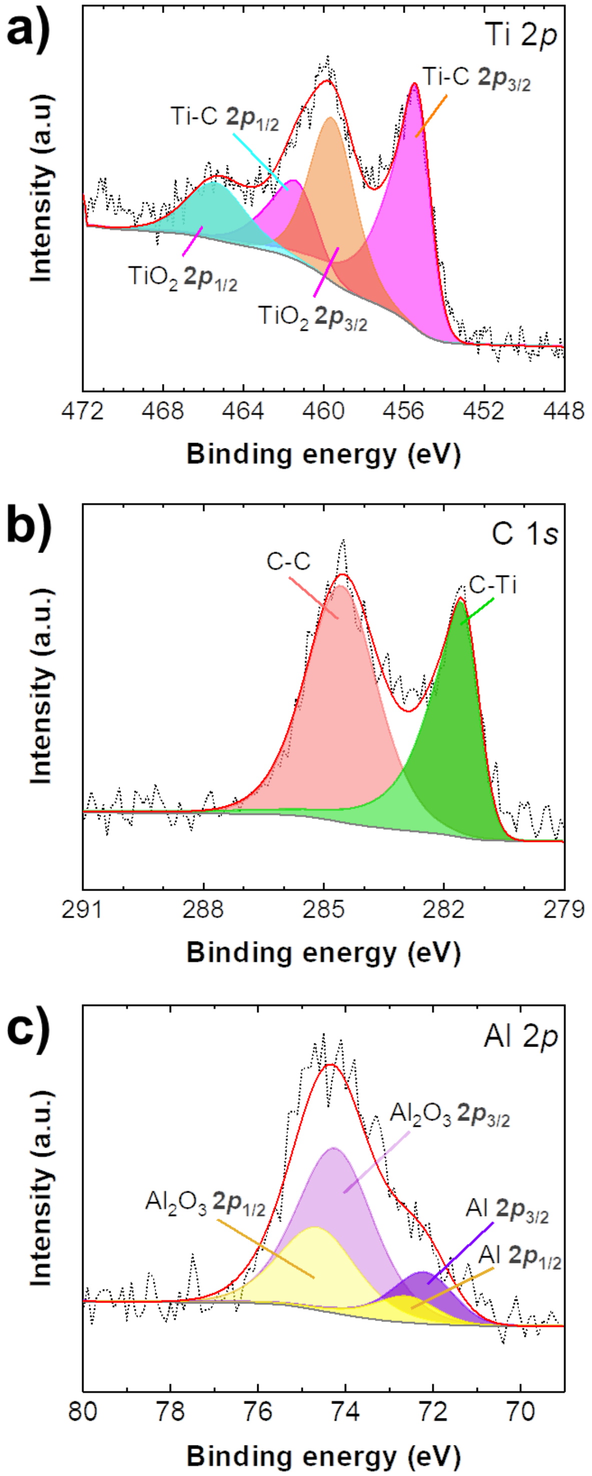

Figure S5. HR-XPS characterization of Ti_3AlC_2 MAX phase: a) Ti 2p, b) C 1s and c) Al 2p core levels. The red straight line represents the fitting, the dark short dotted line represents the experimental result, and the gray straight line represents the background.

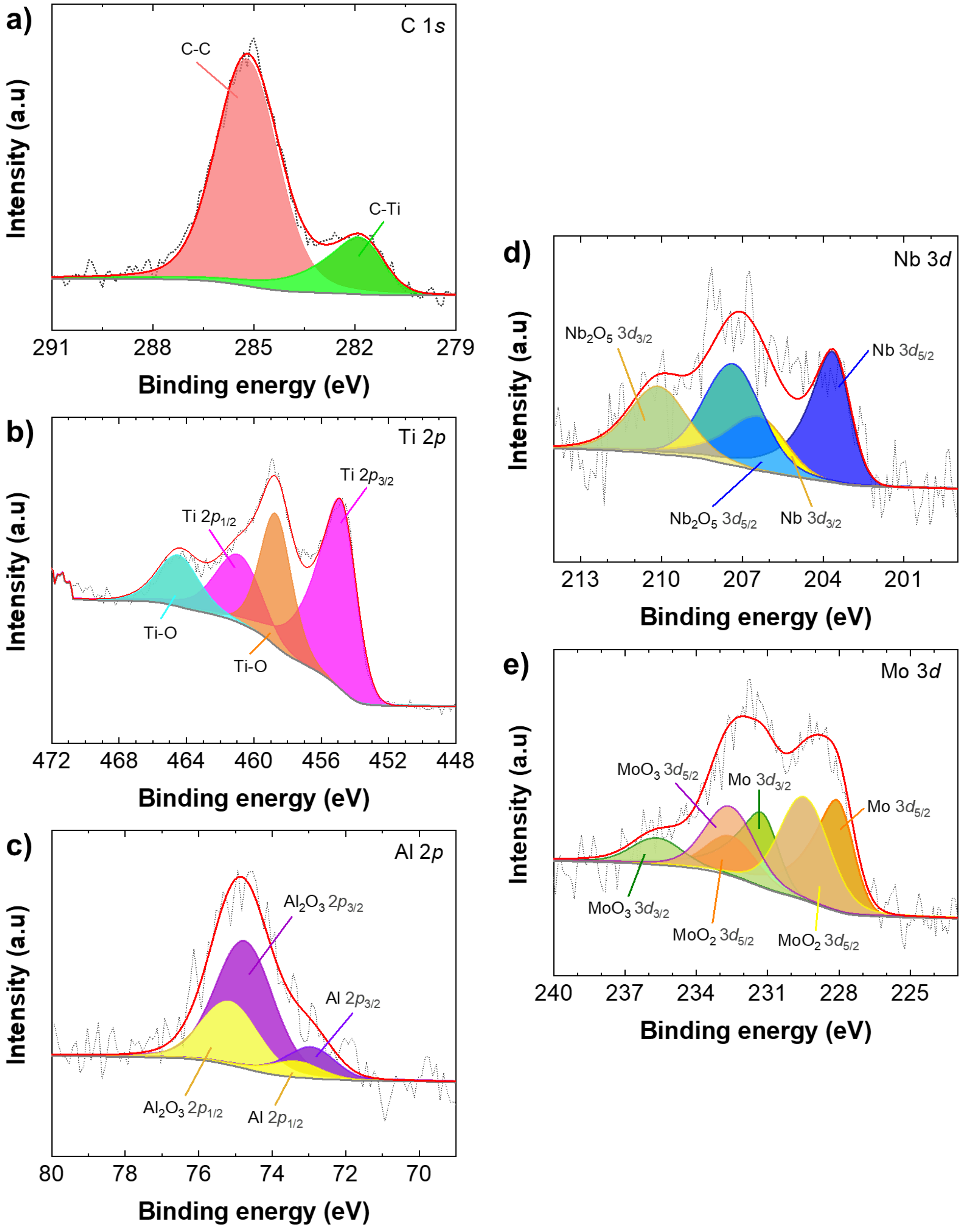

Figure S6. HR-XPS spectra of $Ti_{2.7}Nb_{0.2}Mo_{0.1}AlC_2$ characterized 10 months post-synthesis: a) C 1s, b) Ti 2p, c) Al 2p, d) Nb 3d, and e) Mo 3d core levels. The red straight line represents the fit, the dark short dotted line represents the experimental result and the grey straight line represents the background.

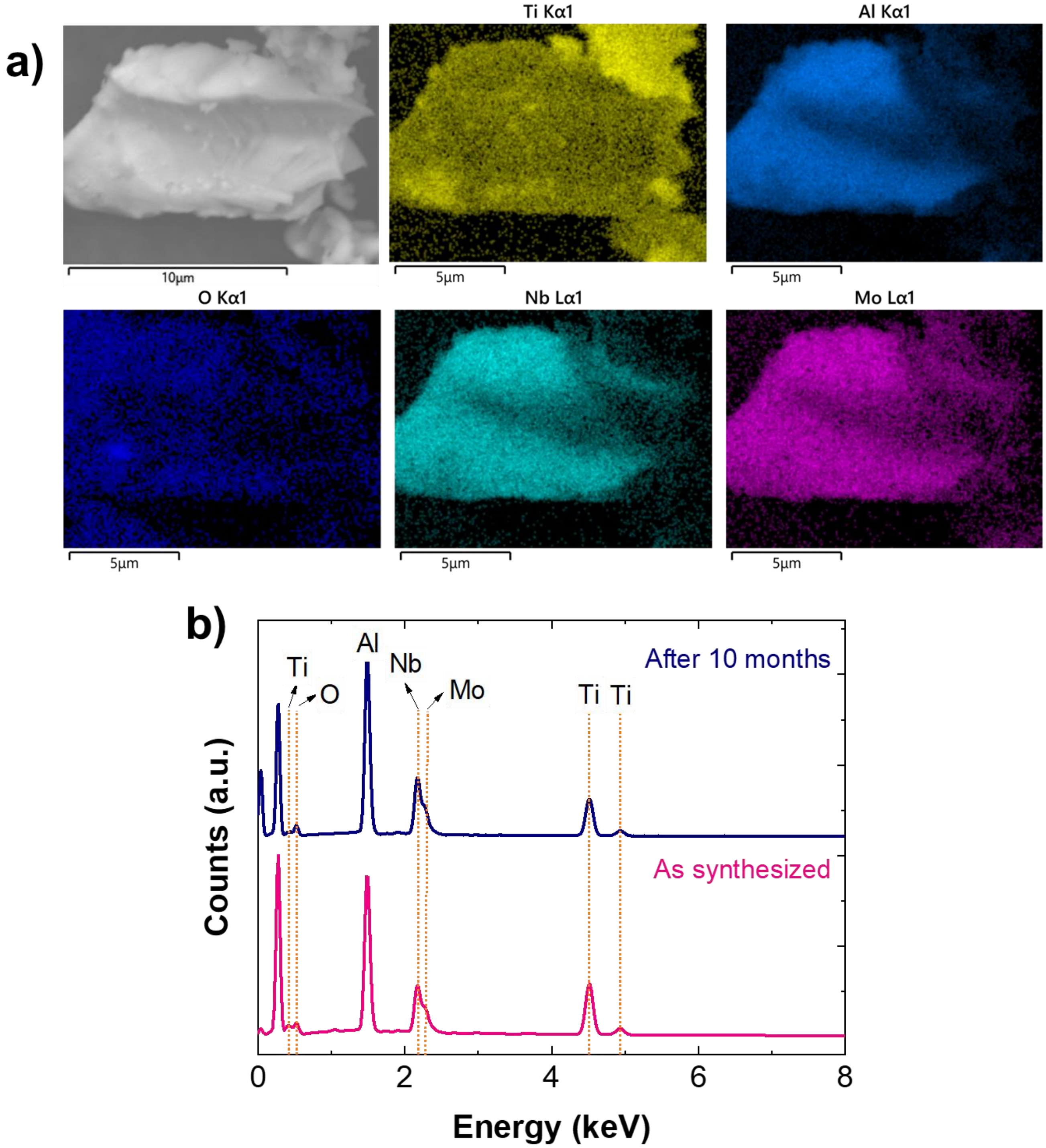

Figure S7. EDS analysis of $Ti_{2.7}Nb_{0.2}Mo_{0.1}AlC_2$ characterized 10 months post-synthesis: a) elemental mapping of the sample demonstrating the uniform distribution of elements. b) Comparative EDS spectra of the as-synthesized sample and the sample analyzed 10 months post-synthesis.

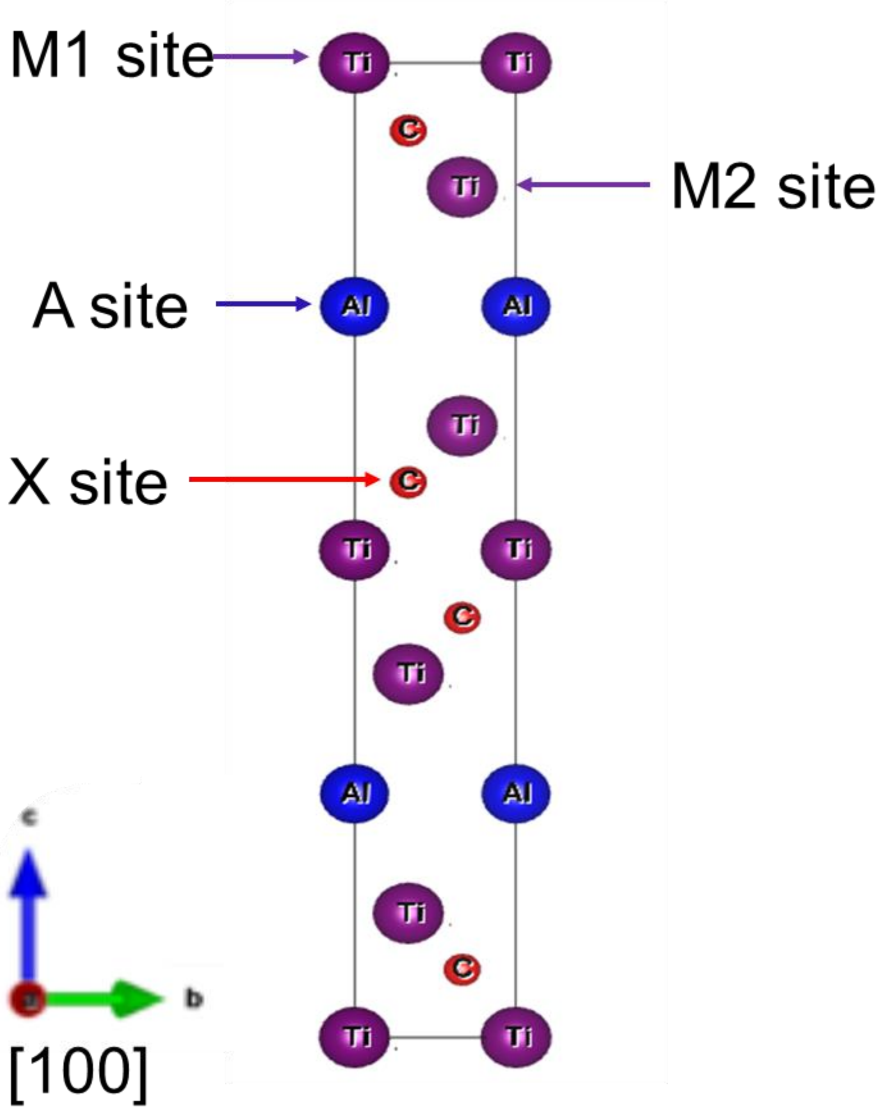

Figure S8. Crystal structure model of Ti_3AlC_2 with M and A site substitutions by Nb and Mo transition metals: $(Ti_{0.6}M1_{0.2}M2_{0.2})_3AlC_2$ (*M1*=Nb, *M2*=Mo) through M site doping, and $(Ti_{0.8}M_{0.2})_3(A_{0.2}Al_{0.8})C_2$ (*M*=Nb, *A*=Mo) through both M and A site doping. The model was constructed using VESTA software.

Table S1. The reaction conditions of synthesized MAX phases by MS^3 method.

MAX phase	Composition of starting materials	Temperature, ºC
Ti_3AlC_2	Ti:Al:C = 3:1.1:1.8	1250
$Ti_{2.7}Nb_{0.2}Mo_{0.1}AlC_2$	Ti:Al:C:Nb:Mo = 3:1.1:1.8:0.2:0.2	

Table S2. XPS data: deconvoluted peaks for synthesized MAX phases.

Peak		Component	Binding energy (eV)		
			**Ti_3AlC_2**	**$Ti_{2.7}Nb_{0.2}Mo_{0.1}AlC_2$**	**$Ti_{2.7}Nb_{0.2}Mo_{0.1}AlC_2$** *10 months post-synthesis*
**Ti 2*p***	**2*p*$_{3/2}$**	Ti–C	454.7	454.9	454.9
		TiO_2	457.4	457.9	458.6
	**2*p*$_{1/2}$**	Ti–C	460.5	460.9	460.9
		TiO_2	463.4	463.9	464.4
**C 1*s***		C–Metal	281.6	281.9	281.9
		C–C	284.5	284.9	285.2
**Al 2*p***	**2*p*$_{3/2}$**	Al	72.1	72.2	73.0
		Al_2O_3	74.2	74.4	74.7
	**2*p*$_{1/2}$**	Al	72.6	72.6	73.3
		Al_2O_3	74.6	74.8	75.1
**Nb 3*d***	**3*d*$_{5/2}$**	Nb–C	n.a.	203.2	203.6
		Nb_2O_5	n.a.	206.9	207.3
	**3*d*$_{3/2}$**	Nb–C	n.a.	205.9	206.4
		Nb_2O_5	n.a.	209.7	210.1
**Mo 3*d***	**3*d*$_{5/2}$**	Mo–C	n.a.	228.1	228.1
		MoO_2	n.a.	-	229.4
		MoO_3	n.a.	-	232.5
		MoO_2	n.a.	-	232.5
	**3*d*$_{3/2}$**	Mo–C	n.a.	231.2	231.3
		MoO_3	n.a.	-	235.6

Table S3. Raman-active modes for synthesized MAX phases. All values are in units of cm^{-1}.

MAX phase	Active mode			
	$\omega_1 [E_{g(1)}]$	$\omega_2\ [A_{1g}]$	$\omega_3\ [A_{1g}]$	$\omega_4\ [E_{g(2)}]$
Ti_3AlC_2	151.5	263.5	408.9	603.5
$Ti_{2.7}Nb_{0.2}Mo_{0.1}AlC_2$	143.4	264.8	386.8	604.8
